# A methodology to estimate the potential to move inpatient to one day surgery

**DOI:** 10.1186/1472-6963-6-78

**Published:** 2006-06-19

**Authors:** Nicolas Gilliard, Yves Eggli, Patricia Halfon

**Affiliations:** 1 Anesthesiology Department, Centre hospitalier universitaire vaudois, 1005 Lausanne, Switzerland; 2 Institut d'économie et de management de la santé, University of Lausanne, César Roux 19, 1005 Lausanne, Switzerland; 3 Institut universitaire de médecine sociale et préventive, University of Lausanne, Rue du Bugnon 17, 1005 Lausanne, Switzerland

## Abstract

**Background:**

The proportion of surgery performed as a day case varies greatly between countries. Low rates suggest a large growth potential in many countries. Measuring the potential development of one day surgery should be grounded on a comprehensive list of eligible procedures, based on a priori criteria, independent of local practices. We propose an algorithmic method, using only routinely available hospital data to identify surgical hospitalizations that could have been performed as one day treatment.

**Methods:**

Moving inpatient surgery to one day surgery was considered feasible if at least one surgical intervention was eligible for one day surgery and if none of the following criteria were present: intervention or affection requiring an inpatient stay, patient transferred or died, and length of stay greater than four days. The eligibility of a procedure to be treated as a day case was mainly established on three a priori criteria: surgical access (endoscopic or not), the invasiveness of the procedure and the size of the operated organ. Few overrides of these criteria occurred when procedures were associated with risk of immediate complications, slow physiological recovery or pain treatment requiring hospital infrastructure. The algorithm was applied to a random sample of one million inpatient US stays and more than 600 thousand Swiss inpatient stays, in the year 2002.

**Results:**

The validity of our method was demonstrated by the few discrepancies between the a priori criteria based list of eligible procedures, and a state list used for reimbursement purposes, the low proportion of hospitalizations eligible for one day care found in the US sample (4.9 versus 19.4% in the Swiss sample), and the distribution of the elective procedures found eligible in Swiss hospitals, well supported by the literature. There were large variations of the proportion of candidates for one day surgery among elective surgical hospitalizations between Swiss hospitals (3 to 45.3%).

**Conclusion:**

The proposed approach allows the monitoring of the proportion of inpatient stay candidates for one day surgery. It could be used for infrastructure planning, resources negotiation and the surveillance of appropriate resource utilization.

## Background

In the last two decades, surgery carried out in outpatient settings has grown much more strongly than inpatient operations [1-3]. Day surgery is generally defined as a non emergency procedure undertaken during the period of a normal working day (not exceeding 12 hours) in hospitals or other facilities set up for this purpose. The concept does not account for minor surgery under local anesthesia usually conducted in physicians' offices [4,5]. The increase of day surgery was driven by innovations in surgical and anesthetic techniques, together with financial incentives and patient expectations. For many procedures, day surgery appears as an effective and efficient approach, offering several advantages to patients as well as surgeons and hospital managers [6,7]. While cost savings have been the primary incentive to day surgery development, the benefits in term of enhanced social and emotional recovery are important: less nosocomial infections, quicker return to mobility, high satisfaction in centers where the outpatient procedure has become standard practice [8,9].

The proportion of surgery performed as a day case varies greatly between countries, however [1,10]. For the same surgical procedure, there are large variations between countries and among hospitals in the same country [5,11,12]. Day surgery is not yet a common practice in many developed countries, with France [1,10,13,14], Italy [1,15], and Switzerland [16,17], suggesting a large potential for growth.

A significant number of scientific studies deal with the safety issues and outcomes of specific surgical interventions performed as day surgery. Although interesting, this literature does not provide a complete overview and therefore cannot be used to define the field of day surgery properly.

Schematically, there are two main approaches to defining a domain: definition in extension, i.e. exemplified by a list of items, and definition in intention, based on explicit a priori criteria [18]. Some extensive lists of procedures have been proposed, mainly for reimbursement purposes [15,19,20]. These lists are useful because they give some evidence of the feasibility of such procedures during stays of less than 24 hours. Their main shortcoming is that they generally reflect local practices.

A definition in intention has been recently proposed by Dexter and al, based on the principle that only surgical procedures with low anesthetic complexity can be performed in ambulatory care [21]. This complexity, defined by the anesthesia workload apart from anesthesia time, is measured by the American Society of Anesthesiologists' Relative Value Guide (ASA RVG), a scale which is updated annually to reflect changes in surgical practice. For instance knee arthroscopy has three ASA RVG units, whereas a prostatectomy or a laparoscopic cholecystectomy has seven and a heart transplant 20. Practically, the authors set the maximum ASA RVG threshold for deciding if a procedure should be performed or not in an ambulatory facility at seven. Such an approach does not depend too much on local practices, but is based on a theoretically coherent point of view. As the ASA RVG is related to the widely used American Medical Association Current Procedural Terminology (CPT) codes, this approach can be compared to most empirical lists published in the USA [22]. Unfortunately, the RVG is not currently used in other countries and its adaptation to ICD-9-CM classification has several shortcomings, probably due to the fact that only frequent interventions have been extensively analyzed [21]. Many procedures performed commonly as one-day surgery are lacking (e.g. hand, foot, testicle, nerve surgery) and several eligible operations cannot be performed routinely in outpatient units (e.g. vaginal reconstruction, excision of anus, skin transplant). Moreover, anesthetic criteria are disputable to delineate day surgery, because some high risk or painful operations are associated with a relatively low anesthetic workload; for instance lyses of peritoneal adhesion (ASA RVG of 6). We think that a definition in intention should be based on the invasion degree of the surgical organ wound. Its immediate consequences, such as blood loss, postoperative pain and difficulty to recover physiological functions, are probably more accurate to determine length of stay than factors related to anesthesia [23].

We therefore proposed a new theoretical model, based on a priori criteria, allowing the setting of a list of procedures qualified for day surgery. The application of this model to all ICD-9-CM surgical procedures was then compared to a consensual list of one day surgery procedures established for Switzerland [20].

The decision to provide ambulatory surgery, or not to do so, does not depend only on the selected procedure but also on coexisting medical conditions, estimating the potential shift from inpatient to outpatient needs to add patient related criteria to surgical criteria. Two countries were compared: Switzerland where there are as yet few financial incentives to an outpatient shift [24] and the USA, where these incentives are stronger [2].

## Methods

### Material

The US source population was the stratified random subsample, known as the Subsample Inpatient Core File, of the Healthcare Cost and Utilization Project Nationwide Inpatient Sample (HCUP NIS) of 2002, a database representing 20% of all US community hospitals [25]. A random sample of one million records was drawn from the Subsample Inpatient Core File for the study (924 hospitals). The Swiss source population included all discharges in 2002 from Swiss acute care hospitals providing data to the Swiss Federal Statistical Office (more than one million records in 259 hospitals). In addition to these data, we used financial claims of hospitals [26] of canton Vaud (Switzerland) to estimate the frequency of the procedures actually performed as day care.

### Setting up the list of procedures qualified for day surgery

Surgical procedures were classified using the International Classification of Disease – 9^th ^revision – Clinical modifications (ICD-9-CM), that is a statutory basis for coding procedures in many developed countries [1]. To be qualified as surgical, procedures required at least incision through the skin or mucous membrane [27]. Thus, aspiration, injection, catheterization, closed reduction of fracture without a bone fixation device were not considered surgical procedures. Procedures on body surfaces requiring only local anesthesia, that can be performed in an office were also excluded. The ICD-9-CM codes of procedures that do not qualify as surgical care are listed in Appendix A. Diagnostic surgical procedures were retained (e.g. biopsy), as well as an endoscopic or endovascular approach. All surgical procedures were classified by four access routes: 1) open access requiring an organ supply (support of circulatory function for instance), 2) other open access (including approach through a small incision of the abdominal or thoracic wall if using an optic device), 3) other approach through integument small incision (without optic device), percutaneous endoluminal access, endoscopic access through a hollow body structure and closed reduction of fracture with fixation device, 4) direct access to a surface region (skin or body cavity through a speculum). Open access procedures were also classified according to three organ sizes and three surgical deed categories. The organ size category depends on the average mass of the operated organic tissue. Fingers and eye structures were considered very small organs; salivary glands and teeth, lymph nodes, nerves, ocular globe, hands and feet were considered small organs; the third category included all other organs. Organ transplantations, amputations and resections, repair and plastic reconstruction (including open fracture reduction and fixation), anastomosis, reimplantation, replacement for organ deficiency were considered highly invasive procedures. Prosthesis or device removal and extraction were considered minimally invasive. All other surgical procedures were considered as moderately invasive.

**Figure 1:**
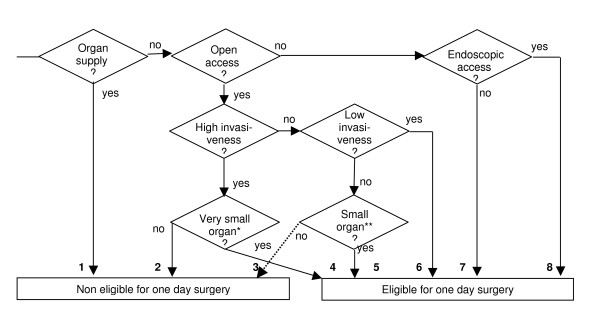
**Algorithmic identification of procedures eligible for one day surgery**. *Very small organs: fingers and eye structures **Small organs: idem + hand, foot, salivary glands and teeth, nerves, lymph nodes.

Open access procedures requiring circulatory support were considered not eligible for one day surgery because of a high mortality risk. The velocity of physiological recovery and the severity of post-surgical pain depend mainly on the extent of organic tissue injured. For this reason, we considered endoscopic procedures as eligible for one day surgery. Similarly, minimally invasive open access procedures were eligible. All highly invasive open access procedures were not qualified as a day case except if performed on a very small organ. Moderately invasive procedures on a very small or small organ could be performed as a day case. Although some obstetrical procedures fulfill all criteria of eligibility for a day case, the duration of deliveries does not allow a same evening discharge and therefore were excluded. The whole assignment scheme of procedures is described in Figure 1.

To set up the reference list, the result of this algorithmic classification was compared to a pragmatic list of procedures eligible for one day surgery edited by a task force in charge of fixing reimbursement rules of day surgery in canton Vaud (Switzerland) [27]. The definition of one day care was a stay of less than 24 hours, without death or transfer to another hospital ("semi-hospitalization"). One day surgery and "semi hospitalization" were not strictly equivalent because the former is generally defined as a same day discharge; however discrepancies were negligible because few surgical "semi-hospitalizations" last beyond midnight. The canton Vaud list has been extensively discussed with representatives of all surgery specialties and formally endorsed by the Swiss All Patients-Diagnosis Related Groups (AP-DRGs) team (Eggli Y. Délimitation de la semi-hospitalisation en Suisse. Lausanne, 2000, unpublished).

All surgical procedures classified as not eligible for one day surgery by the algorithm, but reimbursed more than 10 times as one day care in the canton Vaud during 2002, were added to the reference list, since there was evidence that the usual post operative complications could be managed without hospitalization. All other discrepancies were discussed in depth to set up the reference list. Surgical procedures were considered not qualified for day surgery if they met at least one of the following conditions: undue risk of immediate complications (e.g. excessive blood loss) requiring hospitalization, slow physiological recovery and late ambulation, pain treatment not manageable without hospital infrastructure. Scientific data on the safety of outpatient surgery were collected when necessary, by combining the specific procedure under investigation with the keywords "day care" and "ambulatory surgery" in the Medline database and searching relevant publications by hand in non indexed specialist journals (e.g. Ambulatory Surgery).

### Measuring the potential for moving inpatient surgery to one day surgery in Switzerland

The potential growth of one day surgery has been measured in a set of 67 Swiss hospitals, performing at least 365 therapeutic surgical operations per year, whose discharge data satisfied all the following a priori quality criteria:

a) exhaustive medical coding: more than 95% of hospitalizations had at least one valid diagnosis code; the proportion of minimally invasive therapeutic ICD-9-CM procedures was more than 10%;

b) medical coding accuracy: the proportion of surgical procedure codes with an unspecified surgical deed or operated organ was less than 5% and with an unspecified surgical site less than 30%; the proportion of diagnosis codes with an unspecified organ or disorder was less than 5% and with other unspecified terms was less than 30%;

c) medical coding plausibility: more than 80% of procedures were justified by a disease code (i.e. involving the same organ group, see Appendix C); more than 90% of diseases justifying an operative procedure were indeed associated with a surgical procedure code;

d) other requirement: less than 5% of stays had a missing or unspecified discharge location.

The studied population consisted of all hospitalizations ended in 2002 with a length of stay of more than 24 hours and at least one surgical therapeutic procedure performed. Deliveries and newborns (defined as patients less than one year of age) were excluded from the studied population. Available data were: age, length of stay, ICD-10 codes for diagnosis (up to 10 codes), ICD-9-CM codes for procedures, discharge location, admission mode (urgent or not), some hospital related variables.

A hospitalization was considered as eligible as a one day case if all the following conditions were fulfilled:

• the patient was neither transferred to another hospital, nor died during the stay;

• there was at least one surgical procedure eligible for one day surgery;

• there was no surgical procedure not eligible for one day surgery;

• procedures eligible for a day case, when multiple, affected no more than one organ; see the list of organs in appendix C;

• there was no significant complication of care during the stay [28] and no reopening of a surgical site; operational definition of reopening was the same procedure coded twice or more, or a procedure code indicating a postoperative complication. Admittedly, two same procedures might reflect a bilateral intervention performed subsequently rather than a reopening: we considered such a case as not eligible as a day case.

• there was no diagnosis requiring hospitalization, i.e. classified in the SQLape affection groups [29] listed in Appendix D;

• the length of stay (discharge date - admission date+1) was equal or less than four days.

This screening algorithm was applied in two ways: considering all hospitalizations and only the elective ones.

### USA data

The same screening algorithm was applied to surgical hospitalizations at 376 hospitals performing more than 365 therapeutic surgical procedures per year. For each hospital, the procedure volume per year was estimated by applying the sampling probabilities to data [30].

## Results

### Classification of procedures

The application of the classification algorithm allocated 1412 ICD-9-CM surgical procedures to inpatient care and 890 to one day care. 84 were not considered qualified for day care after review. 55 procedures allocated to hospital care by the algorithm were judged qualified for day care after review. The final list included 861 procedures that could be performed as day care.

Discrepancies between each algorithm sequence and the final classification are shown in Table 1. Procedures that override the algorithm categorization are displayed in Table 2.

### Measuring the potential move to one day surgery in Swiss hospitals

The studied population consisted of 176,345 surgical hospitalizations, of which 135,376 were elective admissions. 27,960 hospitalizations (15.9% of all hospitalizations) and 26,250 elective hospitalizations (19.4% of elective hospitalizations) were found eligible for day care. The rates of elective hospitalizations that could be moved to day care varied between hospitals from 3.0 to 45.3% (median rate 20%, interquartile range 14–26%).

The distribution of elective procedures, the feasibility of which in day care is well supported by literature and identified as eligible by the algorithm, is shown in Table 3. Twenty four interventions represented 88% of the therapeutic interventions performed actually in day care in canton Vaud, one of the few Swiss cantons with financial incentives to promote day care.

Less than 10% of cases were interventions for which feasibility in day care is less supported by literature (Table 4). These interventions represented less than 3% of interventions actually performed as day care in canton Vaud.

Procedures qualified for day care by overriding a priori criteria were performed in 5% of elective hospitalizations; 60% of these hospitalizations were found eligible for day care.

### Measuring the potential move to one day surgery in USA hospitals

The studied population consisted of 193,240 surgical hospitalizations in 376 hospitals, among which 99,267 were elective admissions. Surgical case mix seemed more severe in USA hospitals than in Swiss hospitals, although length of stay distribution was similar (median of five days): more non elective admissions (23% in Switzerland, 49% in USA), higher death rate (1.0% in Switzerland, 2.1% in USA), older patients (average age 54 in Switzerland, 58 in USA). 8540 hospitalizations (4.4% of all candidate hospitalizations) and 5330 elective hospitalizations (5.4% of candidate hospitalizations) were found eligible for day care.

The distribution of elective procedures identified as feasible in day care is shown in Table 5 (ICD-9-CM procedures were grouped in intervention types); these were mostly endovascular heart operations, which in Switzerland are often performed in an inpatient setting. Most interventions often performed in day care in Switzerland (cataract, curettage, circumcision, adenoidectomy, middle ear, implantable vascular device, minor surgery on hands and carpal tunnel release) were almost never found in US hospitals (less than 1% of inpatient surgery eligible for day care). There were two exceptions: skin operations and endoscopic operations on the gastrointestinal tract, which have similar frequencies to those found in Swiss hospitals. Half of skin procedures were plastic operations for obesity (procedure code 8683 associated with a diagnosis code of localized adiposity), which could consist of large volume lipoplasty. Most endoscopic procedures were palliative interventions for malignancy (stomy or dilatation). All other interventions detected by the algorithm were performed in Switzerland more often (or equally often) as inpatient or as day care. Several interventions, still performed as inpatient in Switzerland, were no longer performed in US hospitals: knee arthroscopic surgery, varicous veins surgery, removal of orthopedic material, minor operation on foot, excision of breast lump, testicular surgery.

Procedures qualified for day care by overriding a priori criteria were performed in 4% of elective hospitalizations; only 20% of those hospitalizations were found eligible for day care.

### Other results

The list of procedures eligible for one day surgery was compared to other available lists. Most procedures identified as feasible in day care by the algorithm were already included in the reimbursement list adopted in Switzerland (differences between the two lists generated only 2.3% of the discharges). In similar fashion, these procedures were commonly performed as ambulatory surgery, according to a restricted reference list of 18 intervention groups, adopted in 1997 by the International Association for Ambulatory Surgery to compare the prevalence of ambulatory surgery between OECD member countries [1]; this list contains similar interventions to those listed in Table 3 except 8, 11, 17, 19 and 22. Note that the latter procedures except 17 are regularly performed in canton Vaud (cumulated proportion of 6.9%, see table 3); cystoscopic surgery for urethra structure or bladder tumor (table 3, 17) has been considered suitable for day care for a long time, although it was more frequently performed as inpatient care in canton Vaud and not included in the restricted reference list [31].

Our list of procedures qualified for one day surgery contains none that are physiologically complex [21]. But several procedures considered as not physiologically complex by Dexter et al were not listed by us; the differences concern only highly invasive procedures (resection, plastic reconstruction, etc., see Methods) and procedures justifying an override of our algorithm (Table 2, part 4 to 8).

## Discussion

Although most health professionals and health authorities no longer dispute the benefits of day care surgery in terms of patient outcomes and costs, there is no consensus on the surgery that ought to be performed as day care. Most actual procedures lists are based on adoption curves driven by financial incentives. We propose a comprehensive list, which has strong content validity and allows the routine measure of unnecessary surgical hospitalizations.

The coherence and the content validity of the proposed list are ensured by the classification algorithm, grounded on the surgical route, the complexity of the surgical deed and the mass of injured tissue. Several procedures (see Table 4, 25, 27, 30), although featured in the Swiss reimbursement list, continue to be undertaken as inpatient surgery in many developed countries. However, due to cost containment purposes, there is a growing shift from inpatient to ambulatory settings. For instance, outpatient PTCA, performed for the first time by Kiemerneij and al in 1997, is now considered both safe and feasible for a large part of the routine candidate population [32,33]. Similarly, although many centers routinely admit patients for overnight observation after endoscopic therapy of choledocholithiasis, six hours are sufficient to detect significant complications [34,36].

Only 55 (2%) ICD-9-CM codes were added to form the reference list (Table 2, 1–3). About half these procedures were justified by sufficient case records in canton Vaud and did not give rise to scientific refutation because they are all related to easily accessible organs: knee surgery, operations on hands, operations on eye structures (Table 3, rows 2, 9, 14). The feasibility of most other procedures (Table 3, row 21 and Table 4, rows 28, 29) was well supported by the literature review: new pacing system implantation and replacement [36-39], tubo-ovarian laparoscopic procedures [40-42], vascular bypass for dialysis [43,44]. As regards ambulatory orthopedic surgery (such as hallux valgus, Table 4 row 26), the limitation is often postoperative pain, the most common reason for delayed discharge or unanticipated hospital readmission [45]. However, ambulatory pain management seems increasingly to provide relief equivalent to inpatient management [46].

Most overrides of algorithm criteria leading to procedures exclusion from the reference list were related to a well-documented risk of complications requiring hospital care (Table 2, 4–8). Postoperative hemorrhage, iatrogenic organ injuries or complications requiring reoperation are frequent after endoscopic surgery on sinus, pancreatic ducts and the urinary tract [47-49]. Perforation risks following rigid endoscopy of upper aerodigestive tract (endoscopic larynx procedure like dilation, removal of foreign body) require observation exceeding eight hours [50]. Until recently, prolonged bed rest and overnight hospitalization have been the general practice after non-coronary endovascular procedures. However, the advent of new vascular devices already allows early ambulation. A same evening discharge has been proposed for selected patients after carotid endarteriectomy, since preliminary studies have shown that all severe complications (stroke, neck hematoma) occur very early, allowing restricting observation to eight hours [51]. More studies are nevertheless necessary to make recommendations. Pain management or follow up care require hospital infrastructure or prolonged bed rest after the following procedures: closed reduction of limb fractures, toe amputation, salivary fistula closure, eye evisceration. Postoperative discomfort generally justified hospital care in retinal detachment surgery: limited studies have suggested that selected patients might be treated with ambulatory vitrectomy [52]. Although peri-anal or anal incision can be achieved under local anesthesia, anal surgery (except operation on hemorrhoids) is often performed as an inpatient procedure, due to wound related problems, i.e. pain and discomfort caused by drains or irrigation. Although the advantages of day case surgery have long been established for groin hernias, the repair of umbilical hernia remains an inpatient procedure, probably because the type of repair may be influenced by the size of the defect. However, recent studies have shown the feasibility of most repair of umbilical hernia under local anesthesia as a means of reducing unacceptably long waiting times in some countries [53].

Finally, a few surgical procedures identified which qualified as day cases by the classification algorithm, were neither featured in the Swiss consensual list nor performed as day cases in canton Vaud, and were only discussed in anecdotal literature reports: corneal transplantation [54], large skin grafting [55], reconstruction of external ear [56], testicular surgery [57]. These procedures being associated with an average length of stay greatly exceeding three days they were not retained in the list.

To investigate the use of ambulatory surgery, most studies simply compare with benchmarks the proportion of specific procedures performed on outpatients. This approach is flawed because other criteria than the specific type of procedure performed may limit ambulatory use: the patient's clinical condition, other associated procedures.

The low rate of candidacy for day surgery in USA (about 4%) speaks in favor of the validity of our list of surgical procedures eligible for one day surgery. The high rate of candidacy and the variability among hospitals in Switzerland demonstrate the benefits of monitoring the potential development of one day surgery.

In Switzerland, each canton has its own hospital organization and financing rules. Some cantons (Vaud until the year 2003 for instance) have set strong financial incentives to develop one day surgery (better pay for physicians if surgery was performed in a length of stay of less than 24 hours), while other cantons did not introduce such mechanisms, financing hospitals on a length of stay basis for instance. There is no doubt that differences in resource allocation are probably the main reason for the high variability of rates of candidates for one day care [17].

The study has some limitations that do not invalidate our conclusions, but could serve to further research. First of all, we limited the measure of potential day cases to stays shorter than four days. This choice is grounded on pragmatic considerations, but the limit might be pushed to five or six days, especially in countries having longer lengths of stay. The answer to this question requires a systematic review of medical records by experienced surgeons.

Another question is related to admission circumstances. Day care could also be considered for emergency admissions, especially if they occur in the morning. Restricting the scope of one day treatment to elective referrals is probably a conservative approach. Some emergency admissions could fit all the criteria for elective day care. Most patients with a superficial abscess or minor injuries are indeed treated as inpatients, on daily priority-based emergency lists, with a more or less long pre-operative stay and even a postoperative night in hospital depending of the time of surgery. Some studies have shown the feasibility of running an emergency day surgery service for certain minor surgical emergencies [58]. However, such a procedure would have to be coordinated by a dedicated manager and thus is not widespread.

The exclusion of medical claims of hospitals with poor data quality could be a bias. However, an analysis of such excluded cases showed very similar results.

Our algorithm did not account for patients' residence. Some inhospital day stays could be justified by distance, especially if the patient cannot rely on assistance by relatives. This variable would deserve to be considered when comparing hospital performances, located in different regions (mountain, rural or urban areas). Our algorithm detects hospitalizations that are candidates for one day care. This should not lead us to conclude that all of them should be performed as day surgery. Nevertheless, our results suggest that the rate of candidates should be less than 6%.

## Conclusion

The proposed approach allows the monitoring of the rate of inpatients stay candidates for one day surgery. Mainly based on rational and a priori criteria, the analysis showed large variations of rates between hospitals. The methodology can be used for infrastructure planning, resources negotiation and monitoring of resource utilization.

## Competing interests

Yves Eggli is the author of the SQLape^® ^patients classification system used in this article and its promoter through the SQLape s.à.r.l., Ch. de la Paix 43, CH-1802 Corseaux (Switzerland). The two other authors do not have any competing interests.

## Authors' contributions

Nicolas Gilliard developed the algorithmic identification of procedures eligible for one day surgery, on the basis of a first list established and systematized by Yves Eggli. Patricia Halfon carried out the literature review and the data analysis. All authors contributed equally to the design, to the validation of the proposed method and to the writing of the article.

## Pre-publication history

The pre-publication history for this paper can be accessed here:



## Supplementary Material

Additional file 1 ICD-9-CM codes of procedures that do not qualify as surgical procedures. (Table in Word format, listing by system all ICD-9-CM procedures codes that do not qualify as surgical procedures) Click here for file

Additional file 2 Procedures identifying a postoperative complication. (Table in Word format, listing ICD-9-CM procedures codes identifying surgery for a postoperative complication) Click here for file

Additional file 3 Organs list according to the Latin or Greek (gr) denomination. (Table in Word format displaying the organs list) Click here for file

Additional file 4 Diagnostic categories requiring hospitalization (SQLape groups). (Table in Word format displaying the list of the diagnostic categories requiring hospitalizations, based on SQLape grouper, a new patient classification system, described in more details on ) Click here for file

## Figures and Tables

**Table 1:** Elaboration of the list of surgical procedures eligible for day care

Category of procedure	Number of procedures codes		
	Algorithm application (see figure 1)	Override (see table 2)	Eligible for day care

1. Procedure requiring circulatory support	94	0	0
2. Highly invasive open access procedure, very small organs excepted	605	16	16
3. Moderately invasive open access procedure, small and very small organs excepted	713	39	39
4. Highly invasive open access procedure, very small organs	13	6	7
5. Moderately invasive open access procedure, small and very small organs	143	28	115
6. Minimally invasive open access procedure	52	3	49
7. Endoscopic, endoluminal access, and closed reduction of fracture	383	21	362
8. Direct access procedures	299	26	273

TOTAL	2302*	139	861

*Among 3618 procedures included in the ICD-9-CM classification, 1172 were non surgical; 140 surgical procedures performed on body surface may be undertaken at the physician's office.

**Table 2:** List of surgical procedures overriding algorithm categorization

Category of procedure	ICD-9-CM surgical procedures
*Surgical procedures eligible for day care:*	
1. Procedure requiring circulatory support	No override
2. Highly invasive open access procedure, very small organs excepted:	Plastic operations on hand bone or tendon (7824, 7834, 7854, 7874, 8251, 8252, 8257), Fasciectomy (8344), Plastic operations on peripheral nerves (046, 0474, 0475, 0476, 0479), Repair of orbital wound (1664, 1681), Open reduction with fixation of toe (7938)
3. Moderately invasive open access procedure, small and very small organs excepted	Open reduction without internal fixation of a limb fracture or unspecified bone, excepted femur (7920, 7921, 7922, 7926, 7929), Operations on joints (knee: 7896, 806, 8086, 8096, Wrist: 8012, Elbow: 8042, 8092, unspecified joints: 8010, 8019, 8089, 8090, 8099), Operation on tendons: (8303, 8312), Cranial sinus incision and drainage (0121), Insertion of cardiac pacemaker permanent system (3780, 3781, 3782, 3783, 3785, 3786, 3787), Operations on orbit: (1663, 1665, 1666), Tubo-ovarian procedures (6529, 6592, 6611, 6631, 6632, 6639), Vascular bypass for dialysis (3927, 3942), Unspecified hemorrhage control (3998).
*Surgical procedures not eligible for day care:*	
4. Highly invasive open access procedure, very small organs	Operation on posterior chamber (1471, 1472, 1474), Major reconstruction of finger (8281, 8421, 8422)
5. Moderately invasive open access procedure, small and very small organs	Removal of epithelial growth of anterior chamber (1293), Eye evisceration with implant (1631), Salivary fistula closure (2642), Arthrodesis or open reduction of dislocation of wrist (7983, 8125), foot (7987, 7988, 8111, 8113, 8114, 8115, 8116), Division or excision of non peripheral nerves (cranial: 0401, 0402, 0405, 0441, 0442, 0521, 0522, sympathetic: 0523, 0524, 0525, 0529, 059, others: 0742, 2992, 3191, 3331)
6. Minimally invasive open access procedures	Removal of artificial anus (4976), foreign body from peritoneum (5492), spine theca shunt (0398)
7. Endoscopic, endoluminal access, procedures and closed reduction of fracture	Endosc removal of pancreatic stone: (5294), Implantation of cardiomyo-stimulation system (3767), Closed reduction with fixation of femur (7915), Endovascular repair of non coronary vessels (3971, 3979, 3966, 3972), Percutaneous jejunostomy (4432, 4632), Endoscopic procedure on larynx (3021, 3022), Sinusectomy (2242, 2260, 2263, 2264) Tracheostomy (3121, 3129), Control of nasal hemorrhage by septal graft (2107), Transurethral prostatectomy (6021, 6029), Transurethral removal of ureter obstruction (560)
8. Direct access procedures	Umbilical hernia repair (5341, 5349), Perianal or anal incision or excision (4901, 4902, 4903, 4904, 4993), Reconstruction of external ear (1871, 1872), Corneal transplant (1160, 1161, 1163, 1169), Pedicle skin flap (8670, 8671, 8672, 8673, 8674, 8675), Resection of nose (214), Toe amputation (8411), Testicular repair (6141, 6269, 6299, 6359, 6382).

**Table 3:** Interventions for which feasibility as day care is well supported by literature (Swiss proportions)

Type	Inpatient surgery eligible for day care N = 26 250		Day care surgery in Canton Vaud (2001) * N = 11 889
	%	Cumulated %	%

1. Cataract surgery	19.2	19.2	19.8
2. Arthroscopic surgery on knee	12.9	32.1	3.5†
3. Dilatation and curettage of uterus	10.9	43.0	13.3
4. Tonsillectomy with or without adenoidectomy	6.5	49.5	4.3
5. Hernia repair (inguinal or femoral)	5.9	55.5	1.5
6. Ligation or stripping of varicous limb veins	5.8	61.2	0.3
7. Removal of orthopedic material	4.9	66.1	4.7
8. Minor operations on skin, debridement, graft without flap	3.1	69.2	5.2
9. Other operations on eye structures (mainly squint and glaucoma)	2.8	72.0	3.8
10. Nasal plasty or mucous resection	2.7	74.8	2.5
11. Other hysteroscopic surgery	1.9	76.7	1.8
12. Operation on hemorrhoids	1.9	78.6	0.2‡
13. Excision of a breast lump	1.7	80.3	Not available§
14. Minor surgery on hand (except material removal and carpal release)	1.6	82.0	1.9
15. Adenoidectomy (without tonsillectomy)	1.4	83.4	6.7
16. Orchidopexy-varicocele-hydrocele and deferens surgery (without testicular resection)	1.4	84.8	1.2
17. Endoscopic surgery on bladder and urethra	1.0	85.7	0.7‡
18. Circumcision	0.7	86.4	6.1
19. Endoscopic removal of lesion on gastrointestinal tract (except anus procedures)	0.6	87.0	1.0
20. Operation on middle ear (myringotomy and stapedectomy)	0.6	87.5	1.1
21. Endoscopic tubal sterilization	0.4	87.9	0.5‡
22. Implantable vascular device	0.3	88.2	1.2
23. Minor interventions on teeth, mouth or salivary gland	0.2	88.3	0.3‡
24. Carpal tunnel decompression	0.0	88.4	3.3

*Total*	*88.4*		*85.0*.

because of different procedures coding systems, a strict matching on interventions was not always possible.

† only meniscectomy

‡ less than 11 records in canton Vaud

§ the only available information was excision of an integument lesion without precision on its location.

**Table 4:** Interventions for which feasibility as day care is less supported by literature (Swiss proportions)

Type	Inpatient surgery eligible for day care N = 26 250		Day care surgery in Canton Vaud (2001)^§ ^N = 11 889
	%	Cumulated %	%

25- Percutaneous operations on coronary vessels	3.5	3.5	0.4‡
26- Minor operations on foot (mainly for hallux valgus)	1.6	5.1	0.9‡
27- Percutaneous endovascular ablation of a heart lesion	1.2	6.3	0.2‡
28- Insertion or replacement of a pacemaker	0.8	7.0	0.5‡
29- Arterio venous shunt for dialysis	0.1	7.2	0.1‡
30- Endoscopic surgery on biliary ducts	0.1	7.3	0.4‡

*Total*	*7.3*		*2.5*

§ see the first footnote Table 4 ‡ see the foonote Table 4

**Table 5:** Proportions of interventions detected by the algorithm in USA (only if >= 1%)

Type of interventions	Inpatient surgery eligible for day care N = 5 330		
	N	%	Cumulated %

Percutaneous operations on coronary vessels	2591	48.6	48.6
Insertion or replacement of a pacemaker	639	12.0	60.6
Endoscopic surgery on bladder and urethra	273	5.1	65.7
Hysteroscopic surgery other than dilatation and curettage	226	4.2	70.0
Minor operations on skin, debridement, graft without flap	207	3.9	73.8
Tonsillectomy with or without adenoidectomy	103	1.9	75.8
Percutaneous endovascular ablation of a heart lesion	83	1.6	77.3
Hernia repair	79	1.5	78.8
Endoscopic removal of lesion on gastrointestinal tract (except anus procedures)	72	1.4	80.2
Operation on hemorrhoids	63	1.2	81.4
Endoscopic surgery on biliary ducts	54	1.0	82.4
Minor interventions on teeth, mouth or salivary gland	54	1.0	83.4
Dilatation and curettage of uterus	53	1.0	84.4

*Total*	*4497*	*84.4*	
