# Supplementary material for: A methodology to estimate the potential to move inpatient to one day surgery
Source: BMC Health Serv Res. 2006 Jun 19;6:78. doi: 10.1186/1472-6963-6-78 (PMC1552063; doi:10.1186/1472-6963-6-78)
Supplement: Additional file 1 — ICD-9-CM codes of procedures that do not qualify as surgical procedures. (Table in Word format, listing by system all ICD-9-CM procedures codes that do not qualify as surgical procedures) [file 1472-6963-6-78-S1.doc]

# Additional file 1

**ICD-9-CM codes of procedures that do not qualify as surgical procedures**

# *Cardiovascular system*

0001; 0002; 0003; 3604; 3721; 3722; 3723; 3726; 3727; 3792; 3793; 3797; 3822; 3829; 3891; 3892; 3893; 3895; 3898; 3899; 3992; 3994; 3995; 3996; 3997; 4019; 7538;

# *Digestive system*

4222; 4223; 4229; 4412; 4413; 4419; 4444; 4493 ; 4494; 4512; 4513; 4519; 4522; 4523; 4524; 4528; 4529; 4695; 4696; 4822; 4823; 4829; 4921; 4922; 4923; 4929; 4941; 4942; 4944; 4951; 4952; 4959; 4971; 4991; 4995; 5201; 5213; 5219; 5292; 5297; 5425; 5429; 5491; 5496; 5497; 5498;

# *Ear*

0941; 0942; 0943; 0944; 0949; 1801; 1802; 1809; 1811; 1812; 1819; 184; 2001; 2009; 201; 2031; 2039; 2072; 2094; 2100; 2101; 2102; 2103; 211; 2121; 2122; 2129; 2161; 2171; 2181; 2191; 2200; 2201; 2202; 2219; 2301; 2309; 2311; 2319; 232; 233; 2341; 2342; 2343; 2349; 235; 236; 2370; 2371; 2372; 2373; 240; 2411; 2412; 2419; 2432; 247; 248; 2499; 2501; 2502; 2509; 2551; 2591; 2593; 2594; 2611; 2619; 2641; 2691; 270; 271; 2721; 2722; 2723; 2724; 2729; 2751; 2752; 2761; 2771; 2791; 2792; 280; 2811; 2819; 2891; 290; 2911; 2912; 2919; 2951;

# *Endocrine system*

0601; 0619;

# *Eye*

0801; 0802; 0809; 0811; 0819; 0841; 0851; 0852; 0881; 0891; 0892; 0893; 090; 0911; 0912; 0919; 0951; 0952; 0953; 0959; 0991; 100; 101; 1021; 1029; 105; 1091; 110; 111; 1121; 1122; 1129; 1141; 1142; 1143; 1151; 1175; 1191; 1221; 1229; 1251;1292;1364; 1411; 1419; 1421; 1422; 1423; 1424; 1425; 1426; 1453; 1454; 1455; 1475; 1509; 1621; 1622; 1629; 1691;

# *Female genital organs*

6511; 6519; 6591; 6619; 6691; 6695; 6719; 6811; 6812; 6819; 696; 697; 6991; 6992; 6994; 6995; 6996; 700; 7011; 7012; 7013; 7014; 7021; 7022; 7023; 7024; 7029; 7071; 7109; 7111; 7119; 7121; 7122; 7123; 7171; 720; 721; 7221; 7229; 7231; 7239; 724; 7251; 7252; 7253; 7254; 726; 7271; 7279; 728; 729; 7301; 7309; 7321; 7322; 733; 734; 7351; 7359; 736; 7391; 7392; 7393; 750; 751; 752; 7531; 7532; 7533; 7534; 7535; 7537; 754; 757; 758; 7591; 7592; 7594;

# *Integumentary system*

5422; 8511; 8519; 8551; 8552; 8581; 8591; 8592;

# *Liver and biliary tract*

5011; 5019; 5091; 5093; 5094; 5101; 5102; 5110; 5111; 5115; 5119; 5142; 5143; 5186;

# *Lymphatic and hematopietic organs*

4100; 4101; 4102; 4103; 4104; 4105; 4106; 4107; 4108; 4109; 4132; 4138; 4139; 4191; 4192;

# *Male genital organs*

6119; 6191; 620; 6211; 6219; 6261; 6291; 6292; 6309; 6351; 636; 6370; 6371; 6372; 6384; 6391; 6392; 6393; 6394; 6395; 6411; 6419; 6491; 6492; 6493; 6494; 6496;

# *Musculoskeletal system*

7609; 7619; 7671; 7673; 7675; 7678; 7693; 7695; 7696; 7880; 7882; 7883; 7884; 7885; 7886; 7887; 7888; 7889; 7900; 7901; 7902; 7903; 7904; 7905; 7906; 7907; 7908; 7909; 7940; 7941; 7942; 7945; 7946; 7949; 7970; 7971; 7972; 7973; 7974; 7975; 7976; 7977; 7978; 7979; 8020; 8021; 8022; 8023; 8024; 8025; 8026; 8027; 8028; 8029; 8191; 8192; 8292; 8293; 8294; 8295; 8296; 8329; 8394; 8395; 8396; 8397; 8398; 8441; 8442; 8443; 8445; 8446; 8447; 8601; 8602; 8603; 8604; 8619; 8624; 8626; 8659; 8684; 8692;

# *Nervous system*

0101; 0102; 0109; 0241; 0294; 0295; 0331; 038; 0390; 0391; 0392; 0395; 0480; 0481; 0489; 0531; 0532; 0539;

# *Respiratory system*

310; 3141; 3142; 3148; 3149; 3193; 3194; 3321; 3322; 3323; 3324; 3329; 3332; 3333; 3404; 3425; 3428; 3429; 346; 3491; 3492; 370; 3729; 7881;

# *Urinary system*

5523; 5529; 5592; 5593; 5594; 5595; 5596; 5597; 5598; 5631 ; 5635; 5639; 5693; 570; 5711; 5731; 5732; 5739; 5794; 5795; 5797; 5821; 5822; 5829; 5929; 5972; 598; 5993; 5994; 5995; 6011; 6018; 6019; 6071; 6091; 6092;

# *Miscellaneous non surgical procedures*

0009; 0010; 0011; 0012; 0013; 0014; 0015; 0719;

All codes greater than 8699
