# Supplementary material for: A methodology to estimate the potential to move inpatient to one day surgery
Source: BMC Health Serv Res. 2006 Jun 19;6:78. doi: 10.1186/1472-6963-6-78 (PMC1552063; doi:10.1186/1472-6963-6-78)
Supplement: Additional file 2 — Procedures identifying a postoperative complication. (Table in Word format, listing ICD-9-CM procedures codes identifying surgery for a postoperative complication) [file 1472-6963-6-78-S2.doc]

**Procedures identifying an iatrogenic postoperative complication**

**Reopening of a surgical site**

0123...Reopen craniotomy site

0302...Reopen laminectomy site

0602...Reopen thyroid field wnd

3403...Reopen thoracotomy site

3595...Heart repair revision

3949...Vasc proc revision NEC

5412...Reopen recent lap site

5461...Reclose post op disrupt

**Surgery for post-surgical hemorrhage or hematoma**

287...Hemorr contrl post T & A

3941...Postop vasc op hem contr

3998...Hemorrhage control NOS

4443...Endosc control gast hem

4449...Other control gast hem

5793...Control bladd hemorrhage

6094...Control prostate hemorr

**Surgery for surgery related laceration**

2951...Suture of pharyngeal lacer

3161...Suture of laryngeal lacer

3171...Suture of tracheal lacer

3341...Bronchial lacerat suture

3343...Lung laceration closure

4282...Suture esophageal lacer

4461...Suture gastric lacerat

4671...Duodenal lacerat suture

4673...Small bowel suture NEC

4675...Suture large bowel lacerat

4871...Suture of rectal lacer

5061...Closure liver lacerat

5191...Repair gallbladder lacerat

5581...Suture kidney laceration

5682...Suture ureteral lacerat

5781...Suture bladder lacerat

5841...Suture urethral lacerat

6941...Suture uterine lacerat
