# Supplementary material for: A methodology to estimate the potential to move inpatient to one day surgery
Source: BMC Health Serv Res. 2006 Jun 19;6:78. doi: 10.1186/1472-6963-6-78 (PMC1552063; doi:10.1186/1472-6963-6-78)
Supplement: Additional file 3 — Organs list according to the Latin or Greek (gr) denomination. (Table in Word format displaying the organs list) [file 1472-6963-6-78-S3.doc]

**Organs list - Latin or Greek (gr) denomination**

| Anus Anus praeter Appendix Arteria coronaris Auricula, externus Auricula, internus Auricula, medius Brachium Bucca Camera, anterior Camera, posterior Cerebrum Columna vertebralis Cor Coxendix Cranium Crus Crystallinus Cutis Dens | Diaphragma Digitus Facies Genu Glandula salivaris Hepaticus Hypo & phusis (gr) In, determinare Intestinum crassum Intestinum tenue Larynx (gr) Lien Mamma Manus Medulla spinalis Musculus Nasus Nervus Nodus lymphaticus Oculus | Oisophagos (gr) Orbita Ossium Ourêter (gr) Ovarium Palpebra Pankreas (gr) Pankreas, exo, krinein (gr) Paries abdominis Pedis Penis Peritonaeum Pharynx (gr) Post parturiens Prae parturiens Prostata Pulmo Rectum intestinum Ren Sanguis | Scapulae Sinus Stomachus Supra- & ren Testis Thorax Thuroeidès (gr) Thymum Tonsillae Trachia Tractus bilis Tunica conjunctiva Ulna Urethra Uterus Vagina Valva Vas Vesica Vulva |
| --- | --- | --- | --- |
