# Supplementary material for: A methodology to estimate the potential to move inpatient to one day surgery
Source: BMC Health Serv Res. 2006 Jun 19;6:78. doi: 10.1186/1472-6963-6-78 (PMC1552063; doi:10.1186/1472-6963-6-78)
Supplement: Additional file 4 — Diagnostic categories requiring hospitalization (SQLape groups). (Table in Word format displaying the list of the diagnostic categories requiring hospitalizations, based on SQLape grouper, a new patient classification system, described in more details on ) [file 1472-6963-6-78-S4.doc]

**Diagnostic categories requiring hospitalization (SQLape groups)**

**See www.SQLape.com for detailed description of SQLape groups**

Myocardial infarction (C+sC) ; Major cardiac congenital anomalies (C-aC) ; Heart failure (C-dC) ; Ruptured anevrysm of large vessel (C-dH) ; Peri-, endo- or myo-carditis (C-iC) ; Other thrombosis or embolism (C-oG) ; Thrombosis or embolism of limb (C-oV) ; Acute ischemic heart disease (C-sC) ;Gangrene (C-sV) ; Gastrointest hemorrhage (D-dD) ; Complicated intest diverticula or fistula (D-dI) ; Gastro intestinal specified infection (D-iD) ; Peritonitis (D-iP) ; Malignant neoplasm, intestine (D-mI) ; Malignant neoplasm, esophagus (D-mO) ; Malignant neoplasm,anus & rectum (D-mR) ; Malignant neoplasm, stomach (D-mS) ; Intestinal obstruction (D-oI) ; Diabetes with complication (E-dD) ; Malignant neoplasm, ovary (F-mO) ; Liver abscess (H+iH) ; Liver cirrhosis (H-dH) ; Chronic pancreatitis (H-dP) ; Cholecystitis or angiocholitis (H-iB) ; Acute pancreatitis (H-iP) ; Malignant neoplasm, pancreas (H-mP) ; Inflammatory arthropathy (L-iO) ; Fracture of pelvis (L-tC) ; Skull injury (L-tT) ; Vertebral column injury (L-tV) ; Multiple injury (L-tZ) ; Muscular disorders (L-zM) ; Major intracranial injury (N+tC) ; Degenerative disease of brain (N-dC) ; Extended paralysis (N-dM) ; Cereb hemorrhage (N-hC) ; Meningitis or encephalitis (N-iC) ; Guillain-Barre syndrome (N-iM) ; Malignant neoplasm, brain (N-mC) ; Hydrocephalus (N-oC) ; Cerebrovascular accident (N-sC) ; Transient cerebral ischemia (N-sT) ; Minor intra cranial injury (N-tC) ; Severe upper respiratory infection (O+iO) ; Psychosis & delirium (P-fH) ; Nervosa anorexia (P-zA) ; Cystic fibrosis (R-dK) ; Other respiratory failure (R-dR); Acute respir failure (R-fR) ; Other pulmonary infection (R-iP) ; Influenza or acute bronchitis (R-iZ) ; Malignant neoplam, lung (R-mP) ; Pulmonary embolism (R-sP) ; Chest injury (R-tT) ; Severe infection (S+iZ) ; Acquired immunodeficency syndrome (S-dI) ; Agranulocytosis (S-dL) ; Cachexia (S-dS) ; Deficient anemia (S-fA) ; Metab disease (S-fM) ; Disseminated intravascular coagulopathy (S-fT) ; Other septicemia (S-iS) ; Acute lymphoid leukemia (S-mL) ; Acute myeloid leukemia without complications (S-mM) ; Secondary malignant neoplasm, bone (S-mO) ; Lymphoma, other leukemia or hematopoetic malignant neoplasm (S-mS) ; Other anemia (S-zA) ; Diseases of white blood cells or coagulation (S-zL) ; Skin abscess (T+iS) ; Third degree or extended burns (T+tT) ; Decubitus ulcer (T-dD) ; Skin ulcer (T-dS) ; Extended bullous dermatose (T-iB) ; Inflammatory dermatitis (T-iT) ; Open wound qnd non superficial burns (T-tT) ; Acute nephropathy (U-fR) ; Urinary infection (U-iU) ; Eye infection (Y-iO) ; Eye injury (Y-tO) ; Disease of posterior chamber (Y-zP) ; Infection, not classified elsewhere (Z-iZ) ; Chemotherapy (Z-mC) ; Radiotherapy (Z-mR) ; Injury of kidney or non specified intern organ (Z-tZ) ; Complication, not classified elsewhere (Z-zC) ; Transplant rejection (Z-zG) ; Rehabilitation or palliative care (Z-zR) ; External cause or poisoning (Z-zX).
